# OKN-007 Alters Protein Expression Profiles in High-Grade Gliomas: Mass Spectral Analysis of Blood Sera

**DOI:** 10.3390/brainsci12010100

**Published:** 2022-01-12

**Authors:** Rheal A. Towner, James Hocker, Nataliya Smith, Debra Saunders, James Battiste, Jay Hanas

**Affiliations:** 1 Advanced Magnetic Resonance Center, Oklahoma Medical Research Foundation, Oklahoma City, OK 73104, USA; Nataliya-Smith@omrf.org (N.S.); Debra-Saunders@omrf.org (D.S.); 2 Department of Neurosurgery, Stephenson Cancer Center, University of Oklahoma Health Sciences Center, Oklahoma City, OK 73104, USA; James-Battiste@ouhsc.edu; 3 Department of Biochemistry, University of Oklahoma Health Sciences Center, Oklahoma City, OK 73104, USA; Jay-Hanas@ouhsc.edu

**Keywords:** OKN-007, high-grade-glioma, rat, blood sera, mass spectrometry (MS), protein profiles, extracellular matrix (ECM)

## Abstract

Current therapies for high-grade gliomas, particularly glioblastomas (GBM), do not extend patient survival beyond 16–22 months. OKN-007 (OKlahoma Nitrone 007), which is currently in phase II (multi-institutional) clinical trials for GBM patients, and has demonstrated efficacy in several rodent and human xenograft glioma models, shows some promise as an anti-glioma therapeutic, as it affects most aspects of tumorigenesis (tumor cell proliferation, angiogenesis, migration, and apoptosis). Combined with the chemotherapeutic agent temozolomide (TMZ), OKN-007 is even more effective by affecting chemo-resistant tumor cells. In this study, mass spectrometry (MS) methodology ESI-MS, mass peak analysis (Leave One Out Cross Validation (LOOCV) and tandem MS peptide sequence analyses), and bioinformatics analyses (Ingenuity^®^ Pathway Analysis (IPA^®^)), were used to identify up- or down-regulated proteins in the blood sera of F98 glioma-bearing rats, that were either untreated or treated with OKN-007. Proteins of interest identified by tandem MS-MS that were decreased in sera from tumor-bearing rats that were either OKN-007-treated or untreated included ABCA2, ATP5B, CNTN2, ITGA3, KMT2D, MYCBP2, NOTCH3, and VCAN. Conversely, proteins of interest in tumor-bearing rats that were elevated following OKN-007 treatment included ABCA6, ADAMTS18, VWA8, MACF1, and LAMA5. These findings, in general, support our previous gene analysis, indicating that OKN-007 may be effective against the ECM. These findings also surmise that OKN-007 may be more effective against oligodendrogliomas, other brain tumors such as medulloblastoma, and possibly other types of cancers.

## 1. Introduction

Gliomas can either be astrocytomas, oligodendrogliomas, ependymomas, or mixed neuronal-glial tumors, with grades varying from I (least malignant) to IV (most malignant) as established by the World Health Organization (WHO) [1]. High-grade gliomas (HGGs) consist of grade III and IV tumors. Unfortunately, patient survival for grade IV gliomas is between 16–22 months with current standard-of-care (SOC) treatments [1], which includes surgical tumor resection, and follow up therapies including radiation, chemotherapy (temozolomide or TMZ (the most commonly used)) and possible treatment with bevacizumab (an antibody therapy targeting the vascular endothelial growth factor or VEGF).

Our group (Towner) has been studying the effect of a small molecular weight (MW), anti-inflammatory molecule, OKN-007 (disodium 4-[(tert-butyl-imino) methyl] benzene-1,3-disulfonate *N*-oxide; or 2,4-disulfophenyl-*N*-tert-butyl nitrone or, OKlahoma Nitrone 007), for the past decade as a therapeutic agent against HGGs, particularly grade IV glioblastomas (GBM). OKN-007 is currently in phase II multi-institutional clinical trials for both recurrent and newly diagnosed GBM patients in combination therapy with TMZ and radiation. In preclinical studies, our group found that OKN-007 was very effective in significantly reducing tumor volumes and elevating animal survival in various high-grade, orthotopic, glioma models, including rat C6 [2], F98 [3,4], mouse GL261 [5], and human adult U87 [3] and G55 [6], as well as pediatric patient-derived GBM [7] and patient-derived diffuse intrinsic pontine glioma (DIPG) [8] xenografts. In addition to effects on animal survival and tumor volumes, OKN-007 was found to significantly reduce necrosis in F98 rat gliomas [9]. In the rat F98 glioma model, we also obtained RT-PCR and microarray data that indicated that OKN-007 acted through the transforming growth factor β (TGF-β1) pathway as a master regulator that down-regulated 57 genes connected with the extracellular matrix (ECM) [6]. In the F98 glioma model, OKN-007 was found to decrease cell proliferation and angiogenesis and increase tumor cell apoptosis [3,9]. OKN-007 was also found to decrease the levels of in vivo free radicals [4]. Additionally, we discovered that OKN-007 is able to augment a decrease in tumor cell growth when co-administered with TMZ in a human G55 xenograft model [6].

The rat F98 high-grade glioma model has various characteristics that are closely associated with human HGGs, including an invasive tumor growth pattern and overexpression of RAS, PDGFB, EGFR, cyclin D1, and D2 [10]. The F98 glioma cell line was initially generated via an intravenous (Iv) injection of ethyl nitrosourea (ENU) transplacentally into pregnant (20 days gestation) Fisher 344 rats, and then developed brain tumors following cloning when intracerebrally injected into syngeneic Fisher 344 rat brains [10].

In this study, blood sera from non-tumor and F98 tumor-bearing rats were subjected to mass spectral (MS) analyses to determine if MS-MS-isolated proteins from these three treatment groups varied and could be used to elucidate distinctive protein profiles of interest that could help establish some further insights, or support our gene data, regarding the mechanism-of-action (MOA) of OKN-007 in high-grade gliomas.

## 2. Materials and Methods

### 2.1. F98 Rat Glioma Model

The study was performed in accordance with the Institutional Animal Care and Use Committee at the Oklahoma Medical Research Foundation. A total of 30 rats (Fisher 344, 4–6 months old, 200–300 g, male) were used (7–8 rats per treatment group; non-tumor-bearing rats with or without OKN-007, and tumor-bearing rats with or without OKN-007). F98 cells (10^5^ in 10-μL volume) were implanted intracerebrally using a stereotaxic device (2 mm lateral and 2 mm anterior to the bregma at a 3-mm depth) in Fischer 344 rats (male 200–250 g) [9].

### 2.2. OKN-007 Treatment

OKN-007 was obtained from Ryss Laboratories (Union City, CA, USA) and was administered to the rats through their drinking water (concentration of 0.018% *w*/*v*) [3,9]. OKN-007 was given continuously, beginning 15 days after F98 glioma cell implantations (tumor volumes were ~30–50 mm^3^, as determined by morphological magnetic resonance imaging (MRI)), until the end of the study (when tumor volumes reached ~300 mm^3^, or up to 20 days following treatment) [3,9]. Rats taking normal drinking water were considered as controls. The amount of OKN-007 taken in by either F98 or U87 glioma-bearing rats was established by weighing water bottles each day. Rats were singly housed. Rats were estimated to take in ∼10 mg/kg body weight/day of OKN-007 [3].

### 2.3. Blood Collection and Serum Separation from the Whole Blood

Blood samples (1 mL) were obtained via tail vein from each rat and left to stand for 1 h at 37 °C to allow clotting. Blood samples were then left at 4 °C overnight to allow contraction of the blood clots. Blood clots were carefully loosened from the sides of the glass tubes using a glass pipet, and the serum was subsequently centrifuged at 4000 rpm for 20 min at 4 °C. Each serum sample was removed from the clot by gently pipetting off into a clean tube using a glass pipet [11]. Samples were stored at −20 °C.

### 2.4. ESI-MS

Serum ESI-MS and mass peak analysis were performed as described [12,13,14]. In brief, serum samples were diluted (4 uL sera + 1200 µL of a mixture of 50%methanol, 2% formic acid, and 48% water) and analyzed by ESI-MS on an ADVANTAGE (Thermo Fisher, Waltham, MA, USA) ion-trap mass spectrometer in positive ion mode. The source consisted of a fused silica tip (Polymicro Technologies: Phoenix, AZ, USA) having a 20 micron inside diameter, 90 micron external diameter, 1.75 Kv source voltage; 0.34 µA current, and capillary temp of 195 °C, for each injection. The flow rate was 0.23 µL per minute through an Eldex MicroPro HPLC pumping system. Triplicate loop injected MS spectral data were acquired for 15 min periods. MS signal data were extracted from each file in 1.0 *m*/*z* units.

### 2.5. LOOCV Mass Peak Analysis and Statistics

**Figure 1:**
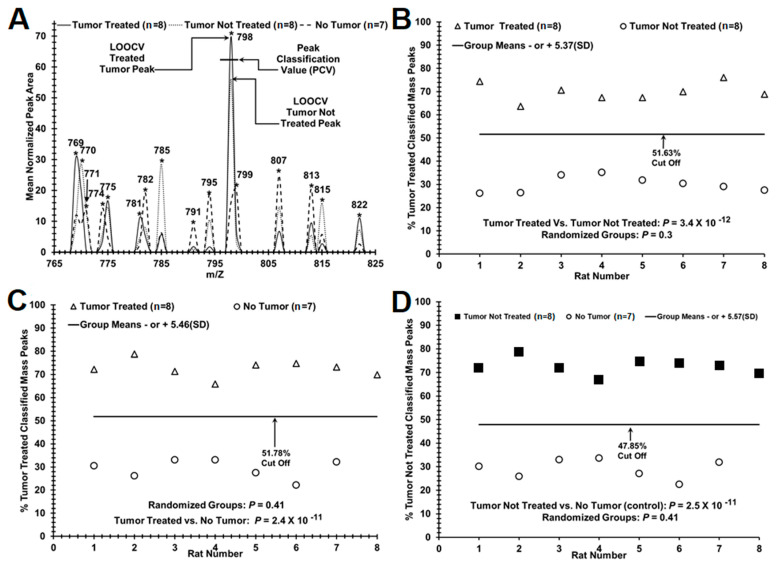
Distinguishing control rodents from treated and untreated brain tumors from each other and from controls using ESI-MS serum mass profiling. ESI-MS (electrospray ionization mass spectrometry) and other procedures were performed as described in the Methods. Panel (**A**) depicts electrospray MS methodology was used to identify, quantify, and classify significant sera *m*/*z* peaks signal into tumor treated (solid line) or tumor not treated (dotted line) peak area descriptors determined by mass peak areas are averages from 8 individual serum samples per category. No tumor controls (dashed line). Panel (**B**) shows a difference in distribution between sera from tumor treated rodents (Δ, open triangles) versus sera of untreated rodents (○, open circles) based on significant “% of tumor treated classified mass peaks”, using the mass peak analyses described in the Materials and in panel A. Panel (**C**), mass peak distribution difference between sera from tumor treated animals (Δ, triangles) and controls. Panel (**D**), mass peak distribution difference between sera from tumor non-treated animals (■, closed squares), and no tumor controls (○, open circles). *p*-values for each group separation are provided. “Cut-offs” are greater than five standard deviations (S.D.) below % mass peaks mean and are used for test metric analysis. * indicates the m/Z molecular mass peak for each major peak identified.

As previously described [12,13,14], post-acquisition MS spectral data processing was performed by locally normalizing each injection data stream set to a total peak intensity value of 100 within a 10 *m*/*z* window along the 150–2000 *m*/*z* observed range. Peaks were identified using standard valley to valley definition and averaged to closest 1 unit *m*/*z* values. Triplicate data for each sample were also averaged to provide a representative spectral peak pattern for each sample. Leave One Out Cross Validation (LOOCV) analysis format was utilized to assess the similarity and significance of peak patterns between the known (treated tumor, tumor not treated, and no tumor) groups, using t-tests (significance designated at *p* < 0.05, one-tailed, unequal variance). LOOCV analysis is used to limit overfitting potential, which is the over-optimistic bias potentially observed when a sample is tested against a set of test variables constructed from a group of samples that also included the sample being tested. The use of the LOOCV procedure helps reduce this bias by serially removing one sample from the dataset and creating a set of test variables from the remaining samples. The variable sample set is then only valid for use against that particular left-out sample. The sample data is then replaced into its proper group, and the next sample is removed, and a new variable set is constructed for it. As graphically presented in Figure 1A, the peak area at each *m*/*z* is compared to the remaining samples. If the left-out sample peak is above the PCV, then that peak is scored with the pathology of the group with the higher mean at that peak; otherwise, the peak is scored with the pathology of the group with the lower mean. When all peaks are scored, the percentage of those peaks scored in each group can be reported on the y axis (see Figure 1B–D). Additionally, a database series is created where treatments groups are evenly mixed (referred to as random groups) to assess the potential for identifying random unrelated data patterns using the same methodology and number of significant peaks. Each sera sample is scored against its respective database by performing the above described LOOCV, analyzing each peak between the 400–2000 *m*/*z* range.

### 2.6. Tandem MS/MS and Bioinformatic Analysis

For tandem MS peptide sequence analysis [14], samples from sera were randomly selected and re-analyzed in the MS ion-trap instrument via selected reaction monitoring (SRM) MS/MS without chromatographic separation of sera. Mass peaks, determined in the ESI-MS positive mode and found to be significant from the LOOCV analysis (*p* < 0.05), were chosen for MS/MS isolation and fragmentation. The significant peaks were between a 500–1100 *m*/*z* range and are a subset of the peaks represented in Figure 1B. Identification of peak proteins was established using SEQUEST Proteome Discoverer 1.0 (Thermo Fisher) employing the “no cleavage” setting on a rat database created through the Discoverer software from an NCBI non-redundant database downloaded on 6 October 2015. Peptides/proteins MS/MS identification from samples involved a cross-correlation value (Xcorr) of 1.8 or better [15]. For Ingenuity^®^ Pathway Analysis (IPA^®^, QIAGEN Germantown, MD, USA www.qiagen.com/ingenuity; accessed on 1 January 2022), associated gene names and the number of identified MS/MS sequences were each imported as base-2 log ratios of untreated tumor sequence “hits” divided by treated tumor “hits”. Detected pathways were manually inspected and verified using Medline/PubMed.

### 2.7. Test Metrics

A test/procedure diagnostic value is defined by sensitivity, specificity, predictive value, and efficiency [16,17]. The sensitivity of the test was determined from TP/(TP+FN), where TP was the number of true positives for disease presence, and FN was the number of false negatives for disease presence. Specificity was calculated from TN/(TN+FP), where TN is the number of true negatives and FP is the number of false positives. Rat brain tumor treated, untreated, and control TP, FP, TN, and FN values were defined using cutoffs of two standard deviations below the mean “% of classified mass peaks patient serum peaks (Figure 1B–D).

## 3. Results

From previous studies, we were able to show that F98 glioma-bearing rats treated with OKN-007 were able to extend animal survival by 9 days (162 equivalent human days; one 6-month-old rat day is an estimated 18 days of human life [18,19,20] following treatment, and significantly decrease tumor volumes ~3-fold [3]. We also previously established that the TGF-β1 pathway was the master regulator that down-regulated 57 genes associated with the ECM [6]. In this study, MS was used to determine the differentiation of protein expressions when comparing non-tumor-bearing and tumor-bearing rats’ blood sera from those treated with OKN-007 (both tumor- and non-tumor-bearing).

Figure 1A illustrate the mean areas of a small number of the significant ESI- MS mass peaks (in *m*/*z* units, mass divided by charge) able to discriminate sera from rodents with drug-treated brain tumors (solid lines), rodents with untreated tumors (dotted lines), and control rodents (dash lines). Major serum mass peak mean areas (higher value) from rodents with treated brain tumors include *m*/*z* 769, 775, 781, and 798, for untreated tumors peaks 770, 785, 815, and peaks 782, 795, and 807 from control animals. This *m*/*z* region is only one of many analyzed (total range 400–2000 *m*/*z*), and a large number of significant peak changes likely is contributing to the brain tumor discrimination ability of this technology. Peak 798 exhibits a Peak Classification Value (PCV) metric area midpoint by which all peaks in the “left in” LOOCV database possess and are similarly assigned and used to classify the group designations (tumor treated, untreated, or control) of the mass peaks found in individual “left out” serum samples. A “left out” peak above this midpoint is allocated to the higher value classifier (tumor treated for this peak), and a “left out” peak below this midpoint is assigned to the lower value classifier (tumor not treated for this peak). In this way, an individual “left out” serum sample is assigned a “% of tumor treated classified mass peaks” or a “% of tumor not treated classified mass peaks”, and that % value is plotted on the y-axis for that patient number in Figure 1B–D. Figure 1B illustrates the application of this LOOCV process using mass peak PCVs for distinguishing serum from rodents with brain tumors treated with a drug from brain tumor untreated animals. When this “% tumor treated classified mass peaks” is plotted versus animal number, a distribution plot is obtained (in which a clear demarcation is observed between tumor treated (triangles) versus tumor untreated (circles). The *p*-value for this distribution difference is very low (10–12 range), and that value becomes non-significant (0.3) when these two sample groups (tumor treated and untreated) are mixed together in a random fashion followed by the same LOOCV mass peak analysis.

The randomized LOOCV database is composed with the randomization of group membership, consisting of the same number of peaks as the original database, and is an attempt to remove the effects of the known pathology, as well as observe the potential separation effects occurring by random, undefined, or unexpected influences. The increase in *p*-value upon randomization is consistent with minimal/reduced over-fitting of the original data set and with the presence of a physiological basis for the group discriminations.

Table 1 provide the LOOCV standard deviations and other metrics of rat brain tumor ESI-MS peak distribution data presented in Figure 1, using the nomenclature from predictive value theory [16,17]. The pathological groups tested in binary fashion are catalogued in the far-left column. The means and their standard deviations (SD) are from the y-axis (% classified peaks) of panels B–D in Figure 1 and are all still well separated and have narrow SD boundaries for each of the groups tested. The *p*-values from the true binary group distribution differences and the mixed randomized binary group distribution differences are indicated. Physiological values in the original distribution differences are shown by the very substantial increases in *p*-values when the groups are randomized. At this stage in the studies with these *n* values, the true positive (test specificity), true negative (test sensitivity), false positive, and false-negative rates are all unit values.

Table 2 exhibit the peptides identified by tandem MS/MS (listed using protein abbreviations) of different protein sequences identified from discriminating sera sample *m*/*z* mass utilized in Figure 1B. MS/MS analysis was performed in sera from all rats in both the untreated tumor (*n* = 8) and the treated tumor (*n* = 8) groups. A subset of mass peaks determined to be significant from the LOOCV analysis distinguishing untreated brain tumor animals from treated brain tumor animals was chosen for MS/MS isolation and fragmentation. In all, 36 significant discriminatory mass peaks at unit Dalton resolution were obtained between a 500–1100 *m*/*z* range and are a subset of the peaks represented in Figure 1, panel B of the separation figure. The top 75 proteins are presented in Table 2. Proteins were considered more likely to occur in serum if they were observed more frequently (minimum two or more unique peptides found in two or more serum samples per group at a cross-correlation (X cor) value of 2.0 or greater. This Table represents a phenotypic “snapshot” of the brain tumor condition in this rat model related to treatment effects and the systemic changes seen in the blood serum. Supplementary Table S1 outlines the ratios of observations used in IPA for the peptides in Table 2 when comparing untreated to OKN-007-treated serum samples.

**Figure 2:**
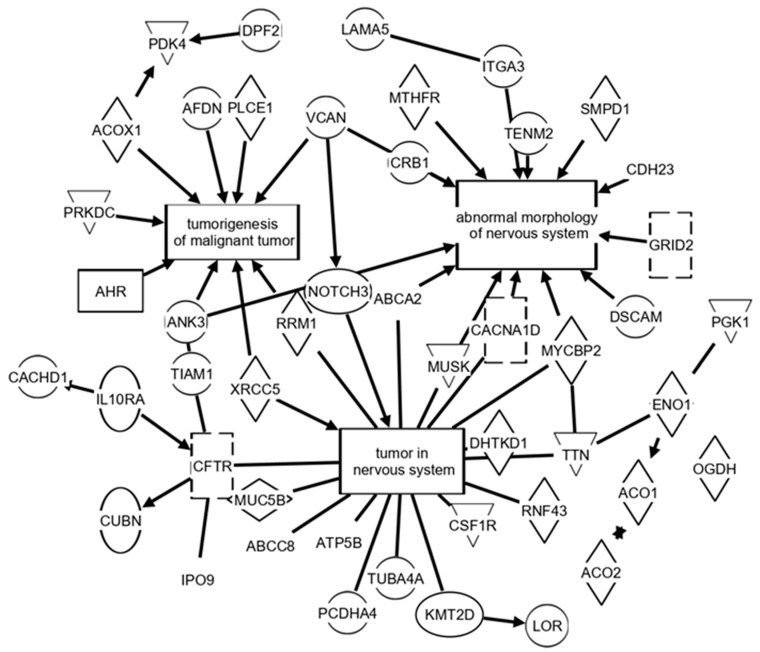
Tumorigenesis and nervous system pathways implicated by Ingenuity^®^ Pathway Analysis (IPA^®^) of serum MS/MS results from untreated vs. treated rat brain tumor model. Values below each protein abbreviation represent the base-2 logarithm [untreated (# hits)/treated (# hits)] utilized by IPA software. Following this format, negative numbers indicate an increase in the number of hits observed in the drug-treated tumor samples compared to untreated tumor samples.

Figure 2 exhibit IPA analysis using the MS/MS results in Table 2 as well as other identified peptides in the “untreated” vs. “treated” peptide analysis from this study. In total, 60% of the proteins indicated in these Figure 2 pathways are included in the “top 75” unique peptide/protein “hits” in Table 2. The remaining 40% are found in the MS/MS results in this study but at lower “number of unique peptides” and “number of MS/MS hits” values. It is noted that zero values suggest a protein that was observed but did not change due to the treatment. Although other pathways could also be identified from these peptides/proteins, the three presented pathways here [tumorigenesis of malignant tumor; abnormal morphology of the nervous system; and tumor in nervous system] represent the simplest representation after duplicating smaller pathways containing the same proteins and similar description/function pathways, were removed. Figure 2 possibly represent a “snapshot” with systemic implications of what could be considered physiological changes related to the treatment of the tumor.

## 4. Discussion

OKN-007, formerly known as NXY-059, was found to have no adverse effects in human safety/toxicity studies [21,22], and we have not seen any adverse effects in any of our preclinical studies. We also have previously shown that OKN-007 is able to cross the blood-brain barrier (BBB) [4], and in fact, temporarily opens up the BBB for a brief 1–2 h period (based on its pharmacokinetics) [23]; however, the mechanism-of-action is currently unknown. It should also be noted that several of the preclinical models for HGGs have “leaky” blood–tumor barriers.

From our previous microarray data assessing genes that were down-regulated by OKN-007 in F98 tumors, compared to untreated tumors, there were 57 genes all associated with the master regulator TGF-β1 [6]. Most of the associated genes were related to the ECM [6]. There were also indications that the mTOR pathway was affected by OKN-007 [6]. Of importance to the proteins discussed below in this study were ITGA1 (integrin alpha1), 2 and 4 (not 3, however), and ADAMTS2 (not 18, however). ITGA1 is a pre-malignant biomarker, which usually fosters therapy resistance and metastatic potential in pancreatic cancer [24]. Notch 3 activation was found to increase the expression of ITGA1 in ovarian cancer cells [25]. ITGA2 is highly expressed in several GBM cell lines [26]. ITGA4 is a metastasis-associated gene [27]. ADAMTS-2 is in the ADAMTS family, and is a procollagen N-proteinase [28]. ADAMTS-2 was found to be overexpressed in gastric cancer fibroblast cells [28]. In addition to the effect of OKN-007 on the ECM as a single agent, our group also established that when OKN-007 is linked with TMZ, it is even more effective in significantly increasing animal survival and decreasing tumor volumes in a G55 high-grade glioma xenograft model [6]. In vitro data also indicated that a combination of OKN-007 with TMZ also resulted in decreasing the cell proliferation of TMZ-resistant human GBM cell lines [6].

From this study, proteins of interest identified by tandem MS-MS that were decreased in sera from tumor-bearing rats treated with OKN-007, compared to untreated, included ABCA2, ATP5B, CNTN2, ITGA3, KMT2D, MYCBP2, NOTCH3, and VCAN. ABCA2 is part of the adenosine triphosphate-binding cassette transporter superfamily and is thought to exert important roles in the transmembrane transport of endogenous lipids, including myelin [29]. The relative expression level of ABCA2 mRNA was found to be significantly higher in oligodendrogliomas compared to anaplastic astrocytomas or GBM [29]. ATP5B has been found to be highly expressed in GBM tumor cells [30]. CNTN2 was found to be highly expressed in oligodendrogliomas [31]. ITGA3 is a cell surface adhesion protein that cooperates with ECM proteins which function in cancer metastasis [32,33]. This finding is somewhat supported by our previous gene data (see above). It was recently shown that brain-specific knockout of the H3K4 methyltransferase MLL4 (a COMPASS (COMplex of Proteins Associated with SET1)-like enzyme, also known as KMT2D) in mice spontaneously induces medulloblastoma [34]. The MYC binding protein 2 (MYCBP2) was found to be a binding partner for the epidermal growth factor receptor (EGFR), which is frequently mutated in various cancers [35]. It is well known that Notch3 activation promotes invasive glioma formation [36]. Versican (VCAN) is a large chondroitin sulphate proteoglycan produced by many tumor cell types, which includes high-grade glioma [37]. The increased expression of particular versican isoforms in the ECM is known to be involved in tumor cell growth, adhesion and migration [37]. Transforming growth factor-beta2 (TGF-beta2) is an essential modulator of glioma invasion, in part via the remodeling of the ECM [37]. Although our previous gene data indicated that TGF-β1 was the master regulator [6], perhaps TGF-β2 also plays an integral role, which would have to be further studied in association with the MOA for OKN-007.

Conversely, proteins of interest in tumor-bearing rats elevated following OKN-007 treatment included ABCA6, ADAMTS18, VWA8, MACF1, and LAMA5. Glioma patients with elevated expression of ABCC8 mRNA were found to have a longer survival [38]. ADAMTS18 is a part of the ADAMTS (A Disintegrin and Metalloproteinase with Thrombospondin motifs) family proteins, which take part in vital roles in cancer progression and metastasis in various cancers [39]. ADAMTS18 was previously found to be downregulated in numerous carcinoma cell lines, which suggested that it could be a tumor suppressor [40]. von Willebrand factor A domain-containing 8 (VWA8) were found to be significantly downregulated in breast cancer brain metastases [41]. Of some concern, MACF1 (microtubule actin cross-linking factor 1) was found to be predominately elevated in grade III-IV astrocytomas and grade IV glioblastoma, however when treated with TMZ, MACF1 is reduced and diminishes GBM cell proliferative capacity [42]. Perhaps when we combine OKN-007 with TMZ [6], the overall effect would be a decrease in MACF1, which we will have to study further in future studies. LAMA5 (laminin alpha5 subunit) is usually associated with cancer invasion/metastasis [43], and we need to further assess the role of serum levels of detected LAMA5 in association with high-grade gliomas, which has not yet been investigated to our knowledge. Of note, in zebrafish xenografts for GBM (U251MG), it was shown that adhesion to LAMA5 was found to inhibit cell invasion [44]. Our in vitro cell migration studies that assessed OKN-007 and OKN-007 combined with TMZ, indicated that G55 GBM cell migration was significantly decreased [6]; however, we did not assess LAMA5 levels, which we would need to study in future studies. Another future study should involve assessing whether TMZ combined with OKN-007 could affect the levels of serum proteins further.

**Figure 3:**
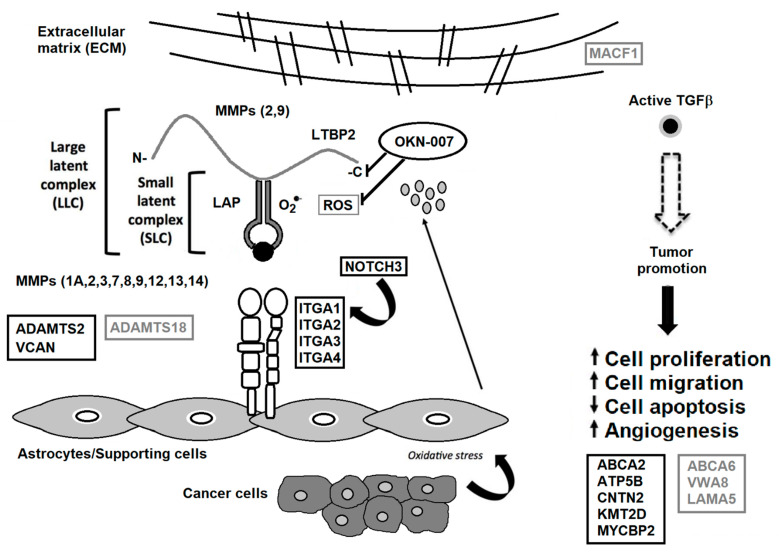
TGF-β associated with the tumor microenvironment. Some matrix metalloproteinases (MMPs) cleave LTBP, which releases latent TGF-β from the extracellular matrix (ECM). Various MMPs activate latent TGF-β through proteolytic cleavage of the latency-associated peptide (LAP), whereas integrins expressed on astrocytes (ITGA1, 2, 3 and 4) bind to the large latent complex (LLC), and activate latent TGF-β through MMP-dependent cleavage of LAP. Integrins (ITGA1, 2, 3 and 4) bind to the LLC and induce conformational changes in the latent complex through contractile action from activated astrocytes. Reactive oxygen species (ROS) produced by activated astrocytes from the induction of oxidative stress from nearby cancer cells may lead to the oxidation of the LAP domain and induce allosteric changes that release mature TGF-β from LAP. The mature (active) form of TGF-β can then bind to its receptor and then turn on tumor-promoting and tumor-suppressive properties. NOTCH3 activates ITGA1 [25], which are both decreased by OKN-007. VCAN is highly expressed in high-grade gliomas [37], which is decreased by OKN-007. ADAMTS2 is highly expressed in some cancers [28], which is decreased by OKN-007. ADAMTS18 is a tumor suppressor [40], which is elevated by OKN-007. During tumor promotion, activated TGF- β led to decreased apoptosis and increased cell proliferation, cell migration, and angiogenesis [45]. OKN-007 is thought to act via LTBP and downregulates several genes associated with the ECM [6], and is also a free radical scavenger [4], resulting in the reversal of the major tumorigenic characteristics, i.e., increases tumor cell apoptosis and decreases cell proliferation, migration and vascular angiogenesis [46]. Other proteins decreased by OKN-007 include ABCA2, ATP5B, CNTN2, KMT2D, and MYCBP2. Other proteins elevated by OKN-007 include ABCA6, VWA8, LAMA5, and MACF1. Serum proteins decreased by OKN-007 are highlighted in black rectangular boxes. Serum proteins elevated by OKN-007 are depicted in gray rectangular boxes. Previous down-regulated genes include *ITGA1*, and *4*, *ADAMTS2*, *MMP 3,* and *12*, as well as several collagen genes (*COL1A1*, *COL3A1*, *COL4A1*, *COL5A1*, *COL6A2,* and *COL7A1*) [6]. Modified from Towner et al. [6] and Costanza et al. [45].

These findings, in general, support our previous gene analysis that indicates that OKN-007 may be effective against the ECM (see Figure 3). These findings also surmise that OKN-007 may be more effective against oligodendrogliomas and possibly other brain tumors such as medulloblastoma, as well as other types of cancers.

## 5. Conclusions

Several proteins were found to be altered in the blood sera of OKN-007-treated F98 tumor-bearing rats. Some of these proteins are highly associated with either gliomas or other cancers, and that OKN-007 can have an advantageous reversal effect in their expressions. There were a few proteins that seemed to be elevated with OKN-007-treatment, which may be of some concern but possibly could be alleviated when combined with other anti-cancer drugs (e.g., TMZ). Overall, the results indicate that when assessing the efficacy of OKN-007 in clinical trials, there may be protein biomarkers of interest that could be simply assessed in blood sera. Blood sera biomarkers used to evaluate therapeutic response against high-grade gliomas are currently non-existent, as far as we know.

## Figures and Tables

**Table 1:** LOOCV Statistics and test metrics for rodent brain tumor and control ESI-MS serum mass profiling.

Test Metrics (Group 1 vs. Group 2, *n* Values)	Mean (SD)	Mean (SD)	True Positive Group 1	False Positive Group 1	True Negative Group 2	False Negative Group 2	*p*-Value True Pathology [Random Groups]
Tumor Treated (8) vs. Tumor Not Treated (8) [ROC area = 1]	0.70	0.30	8/8	0/0	8/8	0/0	3.4 × 10^−12^
	(0.03)	(0.03)	(100%)	(0%)	(100%)	(0%)	[0.3]
Tumor Treated (*n* = 8) vs. No Tumor (control: *n* = 7) [ROC area = 1]	0.73	0.29	8/8	0/0	7/7	0/0	2.4 × 10^−11^
	(0.03)	(0.04)	(100%)	(0%)	(100%)	(0%)	[0.41]
Tumor Not Treated (8) vs. No Tumor (control: *n* = 7) [ROC area = 1]	0.73	0.29	8/8	0/0	7/7	0/0	2.5 × 10^−11^
	(0.03)	(0.04)	(100%)	(0%)	(100%)	(0%)	[0.41]

LOOCV (leave one out cross-validation); SD (standard deviation); ROC (receiver operator characteristic) curve; *p*-value from Student’s *t*-test.

**Table 2:** Peptides/proteins identified by tandem MS/MS to be elevated or decreased in sera from rat brain tumor drug-treated or untreated animals.

**Decreased in Serum from Treated Brain Tumor Animals Relative to Non-Treated Animals**					
**Protein**	**Pathway (IPA)**	**Protein**	**Pathway (IPA)**	**Protein**	**Pathway (IPA)**
ABCA2	B, C, CN, G, MM, MN, T	DDX1	MM	NAT6	
ACO2	C, N	DHTKD1	B, C, CN, G	NOTCH3	B, C, CM, CN, G, N
ADGRE1		DPYSL3	C, MN, N	NSUN6	
AHR	CM, MM, N	FH	CM, MM, N, T	PDE11A	C,
ATP5B	B, CN, G, T	INTS12		PIGS	
CACHD1		IPO9	T	SMG5	T
CES2		ITGA3	C, MM, MN, N, T	TMEM132D	C,
CFTR	C, CN, G, MM, T	KMT2D	C, CM, CN, G	TTBK2	N
CNTN2	MN	LOC108348049		TTN	B, C, CN, G, MM, N
CRB1	MN	LOC690425		VCAN	C, CM, MM, MN, N
CSF1R	C, CM, CN, G, MM, N	MYCBP2	C, CN, G, MM, MN	XRCC5	C, CN, MM, N
CUBN	C, T	MYH3		ZBTB37	
**Increased in Serum from Treated Brain Tumor Animals Relative to Non-Treated Animals**					
**Protein**	**Pathway (IPA)**	**Protein**	**Pathway (IPA)**	**Protein**	**Pathway (IPA)**
ABCA6		GRID2	MM, MN, N, T	MUC16	
ABCC8	CN, MM, N, T	HADHA	MM, N	MUC19	
ACO1	C, T	LAMA5	C, CM, N	MUSK	B, CN, G, MM, MN, N
ACOX1	C	LOC102554371		PAFAH2	N
ADAMTS18	C,	LOC103691264		PCDHA4	B, C, CN, G
ADGRF5	C, CM, MM	LOC685544		PCYT1A	MM, T
AMACR	MM, N	LOR	C,	PLCE1	C, N
CACNA1D	C, CM, CN, MN, T	MACF1	MM	PTPN22	N
CRYBG3		MCM3AP	MM, T	REV3L	MM, N
DHRSX	C,	MFSD12		TBC1D23	
DSCAM	C, MM, MN	MICAL2	C,	TENM2	C, MN
EEF1AKMT1		MRVI1	MM	TUBA4A	
EXNEF	C,	MTTP		VWA8	

Pathways were identified by Ingenuity Pathway Analysis (IPA) software; abbreviations: B (brain astrocytoma); C (cancer of cells); CM (cell movement of tumor cell lines); CN (central nervous system tumor); G (gliomatosis); MM (morbidity or mortality); MN (morphology of nervous system); N (necrosis); T (transport molecule). Proteins indicated as increased in treated were identified more often in the treated samples than in the untreated samples by MS/MS analysis of peaks identified to be significant by the LOOCV analysis.

## Data Availability

Data will be provided from the corresponding authors upon reasonable request.

## Supplementary Materials

The following are available online at https://www.mdpi.com/article/10.3390/brainsci12010100/s1, Table S1: Ratio of observations used in IPA.
