# Supplementary material for: OKN-007 Alters Protein Expression Profiles in High-Grade Gliomas: Mass Spectral Analysis of Blood Sera
Source: Brain Sci. 2022 Jan 12;12(1):100. doi: 10.3390/brainsci12010100 (PMC8773900; doi:10.3390/brainsci12010100)
Supplement: Supplementary file 1 [file brainsci-12-00100-s001.zip › brainsci-1543361-supplementary.pdf]

Table S1. Ratio of observations used in IPA.

protein name	-log2 ratio (untreated) / (treated)		protein name	-log2 ratio (untreated) / (treated)
REV3L	-4.954		ZBTB37	4.585
CACNA1D	-4.17		TTBK2	4.524
TBC1D23	-3.907		IPO9	4
GRID2	-3.807		SMG5	3.585
ACOX1	-3.7		DDX1	3.459
DSCAM	-3.585		MYCBP2	3.322
LOR	-3.322		AHR	3.17
ADGRF5	-3.17		INTS12	3.17
MACF1	-3.17		ATP5B	2.807
MFSD12	-3.17		CFTR	2.807
PLCE1	-3.17		TMEM132D	2.807
HADHA	-3.087		TTN	2.585
AMACR	-2.807		NOTCH3	2.503
MCM3AP	-2.807		LOC690425	2.415
PTPN22	-2.807		PIGS	2.322
TUBA4A	-2.807		XRCC5	2.322
MUC16	-2.663		DHTKD1	1.874
ACO1	-2.115		FH	1.766
LOC685544	-1.874		LOC108348049	1.737
CRYBG3	-1.585		ABCA2	1.415
MICAL2	-1.222		PDE11A	1.379
PCYT1A	-1.222		CNTN2	1.222
MRVI1	-1.138		DPYSL3	1
LOC102554371	-1.07		MYH3	1
ABCA6	-1		ADGRE1	0.737
ADAMTS18	-1		CES2	0.737
LOC103691264	-0.822		CRB1	0.737
VWA8	-0.807		NAT6	0.737
ABCC8	-0.737		VCAN	0.737
DHRSX	-0.737		ACO2	0.617
EEF1AKMT1	-0.737		CACHD1	0.485
EXNEF	-0.737		NSUN6	0.415
LAMA5	-0.737		CUBN	0.322
MUC19	-0.737		KMT2D	0.322
MUSK	-0.737		CSF1R	0.263
PCDHA4	-0.737		ITGA3	0.263
MTTP	-0.415			
PAFAH2	-0.322			
TENM2	-0.322			
